# Association of Socioeconomic Position, Prostate-specific Antigen, and Age with Observation in Low-risk Prostate Cancer Patients in Switzerland

**DOI:** 10.1016/j.euros.2025.12.009

**Published:** 2025-12-30

**Authors:** Thomas Paul Scherer, Dominik Menges, Uwe Bieri, Lea Wildisen, Katharina Staehelin, Florian Alexander Schmid, Basil Kaufmann, Daniel Eberli, Sabine Rohrmann, Cédric Poyet

**Affiliations:** a Department of Urology, University Hospital Zurich (USZ), University of Zurich (UZH), Zurich, Switzerland; b Epidemiology, Biostatistics and Prevention Institute (EBPI), University of Zurich (UZH), Zurich, Switzerland; c Department of Medical Epidemiology and Biostatistics (MEB), Karolinska Institutet (KI), Stockholm, Sweden; d Department of Surgery, Division of Urology, Cantonal Hospital Baden, Aargau, Switzerland; e National Institute for Cancer Epidemiology and Registration (NICER), Zurich, Switzerland; f National Agency for Cancer Registration (NACR), Zurich, Switzerland; g Cancer registry of the Cantons Zurich, Zug, Schaffhausen and Schwyz, University of Zurich, Zurich, Switzerland; h Department of Urology, City Hospital Triemli of Zurich, Zurich, Switzerland

**Keywords:** Active surveillance, Watchful waiting, Observation, Overtreatment, Prostate cancer, Socioeconomic position

## Abstract

**Background and objective:**

Observation remains the recommended management approach for low-risk prostate cancer (PCa), aiming to balance oncological control and avoidance of overtreatment. This study investigated the use of observation and its association with patients’ socioeconomic position (SEP), age, and prostate-specific antigen (PSA) level on treatment choice in Switzerland.

**Methods:**

This cohort study analyzed Gleason score 6 PCa diagnoses in 2020 and 2021 from the Swiss National Agency for Cancer Registration dataset. Variables included age, PSA value, residence, and treatment codes. Municipality-based SEP deciles were linked to patients. Multivariable regression assessed the associations between SEP and observational management.

**Key findings and limitations:**

Of 4296 men, 2876 (65.4%) received observational management, 792 (18.0%) underwent active treatment, and management was unknown in 728 (16.6%). Compared with men from low SEP areas, those from middle (odds ratio [OR] 1.11, 95% confidence interval [CI]: 0.92–1.35) and high SEP (OR 1.29, 95% CI: 1.06–1.58) areas had higher odds of observation. Men aged 60–70 yr (OR 1.53, 95% CI: 1.24–1.89) and >70 yr (OR 2.10, 95% CI: 1.68–2.62) were more likely to undergo observation than those aged <60 yr. PSA 5–10 ng/ml (OR 0.67, 95% CI: 0.55–0.82) and >10 ng/ml (OR 0.60, 95% CI: 0.46–0.78) were associated with lower odds of observation compared with PSA <5 ng/ml.

**Conclusions and clinical implications:**

Most men diagnosed with localized low-risk PCa in Switzerland underwent observational management as the primary strategy in 2020 and 2021. However, at least 18% of men still received active treatment. Lower SEP, younger age, and higher PSA values were risk factors for active treatment within low-risk PCa patients.

**Patient summary:**

We looked at how men in Switzerland with low-risk prostate cancer were treated in 2020 and 2021. We found that most men chose observation instead of immediate treatment, but men with lower socioeconomic position, younger age, or higher prostate-specific antigen levels were more likely to have active treatment. This suggests that there are opportunities to reduce unnecessary treatment for certain patient groups.

## Take Home Message

Most low-risk prostate cancer patients received observational management, but lower socioeconomic position, younger age, and a higher prostate-specific antigen level were associated with higher odds of active treatment. Further work is needed to understand the barriers to active surveillance uptake in low-risk prostate cancer patients.

## 1 Introduction

It is well established that prostate cancer (PCa) of low risk, categorized as PCa with Gleason score 6, exhibits a highly favorable long-term biological profile, characterized by very low morbidity and mortality [1], [2]. Thus, it is nowadays evident that observation is the preferred management strategy, as active treatment would lead to severe overtreatment in most of these men [3]. Based on life expectancy, active surveillance (AS) is recommended for patients with expected survival of >10 yr, while watchful waiting (WW) is appropriate for those with shorter life expectancy (≤10 yr) [3]. AS has been shown to delay or completely avoid unnecessary local curative treatments successfully. Thereby, it spares patients from unnecessary morbidity due to treatment-related adverse effects, while long-term oncological outcomes are comparable with those of immediate active treatment [3], [4], [5]. Despite the clear benefits, its uptake has been shown to vary [6], [7], [8], [9].

The influence of socioeconomic position (SEP) on the probability of undergoing AS is not well understood, as financial incentives may affect treatment decisions and vary depending on the health care system [7], [8], [9]. In addition, it is known that prostate-specific antigen (PSA) level and age are important factors influencing treatment decisions [10].

Recent registry data from the Canton of Zurich in Switzerland showed poor uptake of observation in men with low-risk PCa [11], but national data are currently lacking. This study aimed to analyze the current use of observation at the national level and to assess whether SEP, age, and PSA are associated with its use in Switzerland.

## 2 Patients and methods

This cohort study based on routine cancer registration data from Switzerland was conducted using the data collected by cantonal cancer registries and provided by the Swiss National Agency for Cancer Registration (NACR). All men diagnosed with low-risk PCa in 2020 and 2021 were extracted from the NACR dataset. Low-risk PCa was defined as PCa with a Gleason score of ≤6 [12]. Individual data used in our analysis included TNM stage, Gleason score, PSA level, age group, municipality of residence, and the primary planned treatment, which was coded using the Swiss Classification of Surgical Interventions—Codes (CHOP-Codes) adapted for cancer registration. All men with a Gleason score of ≥7, missing information on Gleason score, N1 stage, M1 stage, or ≥T3 stage, as well as those diagnosed by radical cystoprostatectomy, receiving systemic treatment, or with a PSA value of ≥40 ng/ml were excluded from the analysis. Although some patients with Gleason score 7 qualify for observation, data on the extent of Gleason pattern 4 were unavailable, preventing this distinction. As PCa with Gleason score 6 metastasizes rarely, cases with N1, M1, or ≥T3 disease were excluded likely due to undersampling or registration error. Similarly, PSA >40 ng/ml was considered indicative of possible metastasis or data error. Patients diagnosed incidentally at cystoprostatectomy were excluded, as they had already received treatment and could not be assigned to the observation or prostatectomy groups. Based on the CHOP-Codes, the treatments were categorized into observation (ie, AS or WW), active treatment (ie, radical prostatectomy, radiotherapy, brachytherapy, or focal therapy), and unknown. The list used for treatment categorization is provided in Supplementary Table 1.

To determine the SEP of the men, data from the Swiss neighborhood index of SEP (Swiss-SEP) were used [13]. The Swiss-SEP is derived from the national house-to-house census conducted in Switzerland in 2000. This index categorizes Switzerland into about 1.54 million distinct neighborhoods, which are defined by four aspects: financial status, educational level, employment status, and living conditions. Each neighborhood is defined by a cluster of ∼50 closely situated households. Owing to the limitation that the NACR dataset includes only men's municipalities of residence, Swiss-SEP households were matched to municipalities using public data from the Swiss Federal Office of Topography [14] to obtain an average SEP for each municipality. Then, municipalities were categorized into SEP deciles based on national mean SEP distributions weighted by the municipalities’ population size. Municipality-level SEP deciles were then matched with the municipality of residence for each patient by NACR, and an anonymized dataset without residence information was obtained for the analysis. Last, the SEP for each patient was categorized as low, middle, and high by using SEP tertiles based on the deciles obtained from the dataset. As SEP deciles across municipalities underlie regional variations, SEP terciles were calculated within each region.

Descriptive statistics were utilized to summarize baseline characteristics. Univariable and multivariable logistic regression analyses were conducted to analyze the associations between SEP, age, and PSA and the utilization of observational treatment versus active treatment, excluding cases with unknown treatment. The regression models included the following predefined covariates (all categorical): SEP tertiles (low, middle, and high), age group (<60, 60–70, and >70 yr), PSA level (0–5, 5–10, and >10 ng/ml and missing PSA), and regions (Lake Geneva Region, Midland, Northwestern Switzerland, Eastern Switzerland, Ticino, Central Switzerland, and Zurich). Odds ratios (ORs) with corresponding 95% confidence intervals (95% CIs) were estimated as effect estimates across all regression analyses. Wald tests were used to compute *p* values from model coefficients and their respective variance, with a significance level of *α* < 0.05 defined as statistically significant. No *p* value adjustment for multiple hypothesis testing was performed.

Several sensitivity analyses were conducted to test the robustness of our findings. In a first sensitivity analysis, we omitted region from the multivariable regression model due to potential collinearity with SEP, for which terciles were regionally adapted in the primary model. In a second sensitivity analysis, we used SEP tertiles calculated for the whole country, adjusting for age group and PSA category in the multivariable regression model. Furthermore, as a PSA value of >20 ng/ml excludes patients from AS but not from WW, we performed a third sensitivity analysis excluding patients with PSA >20 ng/ml. Additionally, a fourth sensitivity analysis excluding patients older than 75 yr was performed, as life expectancy of at least 10 yr is recommended to be eligible for AS. Last, to allow additional judgments about the group with unknown treatment, which was omitted from the main regression analyses, we conducted a fifth sensitivity analysis using two multivariable logistic regression models for the associations of SEP, age, and PSA with unknown treatment, compared with observational and active treatment (additionally adjusting for region).

All analyses and data visualizations were performed using Stata 18 (StataCorp., College Station, TX, USA). No approval from the Ethical Committee of the Canton of Zurich was necessary for this work as the use of anonymized cancer registration data does not fall under the Swiss Human Research Act (KEK No. 2023-01126).

## 3 Results

In 2020 and 2021, a total of 4697 men were registered with low-risk PCa in Switzerland. Of these patients, 301 were excluded based on the predefined criteria: 23 with M1, 73 with N1, and 19 with T3 disease. In addition, 81 patients who had received androgen deprivation therapy, 88 who had been diagnosed incidentally following radical cystoprostatectomy, and 17 with PSA ≥40 ng/ml were excluded. After applying the exclusion, 4396 men were used for the final analysis, of whom 724 (16.5%) were younger than 60 yr, 1773 (40.3%) were aged between 60 and 70 yr, and 1899 (43.2%) were older than 70 (Table 1). PSA values were available for 3382 men, with a median PSA value of 5.9 ng/ml (interquartile range: 4.3–8.3 ng/ml).

**Table 1:** Clinical characteristics of men with Gleason score 6 prostate cancer, stratified per chosen management strategy

	Observation	Active treatment	Unknown	Overall
	(*n* = 2876)	(*n* = 792)	(*n* = 728)	(*n* = 4396)
Age category (yr), *n* (%)				
<60	449 (15.6)	195 (24.6)	80 (11.0)	724 (16.5)
60–70	1199 (41.7)	340 (42.9)	234 (32.1)	1773 (40.3)
>70	1228 (42.7)	257 (32.4)	414 (56.9)	1899 (43.2)
PSA category (ng/ml), *n* (%)				
<5	852 (29.6)	196 (24.7)	144 (19.8)	1192 (27.1)
5–10	1185 (41.2)	377 (47.6)	113 (15.5)	1675 (38.1)
>10	357 (12.4)	117 (14.8)	41 (5.6)	515 (11.7)
Missing PSA	482 (16.8)	102 (12.9)	430 (59.1)	1014 (23.1)
Socioeconomic position, *n* (%)				
Low	1064 (37.0)	319 (40.3)	264 (36.3)	1647 (37.5)
Middle	926 (32.2)	258 (32.6)	223 (30.6)	1407 (32.0)
High	886 (30.8)	215 (27.1)	241 (33.1)	1342 (30.5)
Region, *n* (%)				
Lake Geneva Region	516 (17.9)	188 (23.7)	259 (35.6)	963 (21.9)
Midland	759 (26.4)	127 (16.0)	97 (13.3)	983 (22.4)
Northwestern Switzerland	400 (13.9)	119 (15.0)	75 (10.3)	594 (13.5)
Eastern Switzerland	496 (17.2)	129 (16.3)	104 (14.3)	729 (16.6)
Ticino	57 (2.0)	32 (4.0)	49 (6.7)	138 (3.1)
Central Switzerland	230 (8.0)	90 (11.4)	98 (13.5)	418 (9.5)
Zurich	418 (14.5)	107 (13.5)	46 (6.3)	571 (13.0)

PSA = prostate-specific antigen.

**Fig. 1:**
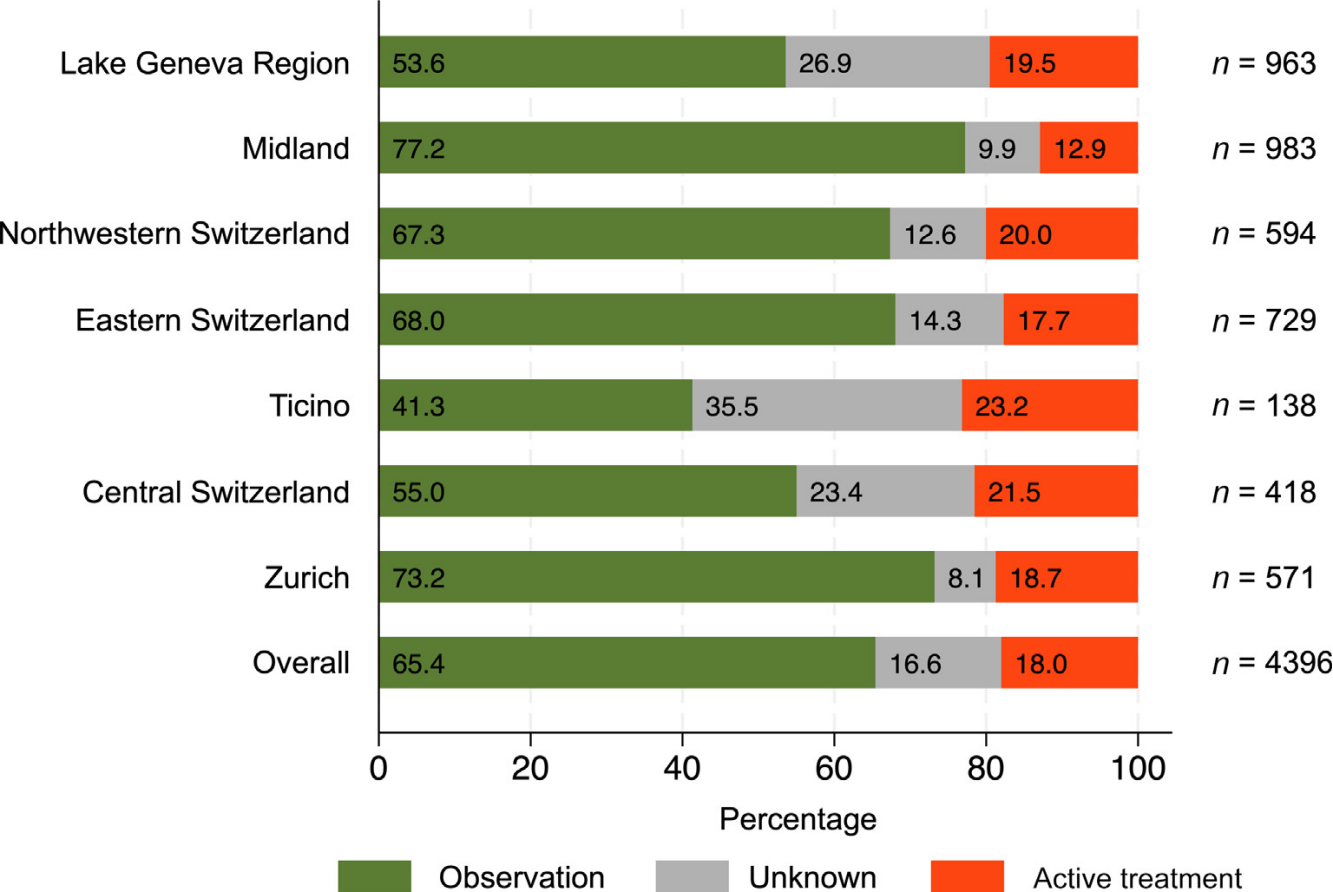
Distribution of chosen treatment among men with Gleason score 6 prostate cancer across Swiss regions. The bars represent the proportion of patients managed with observation (green), unknown treatment (gray), or active treatment (red). The number of patients per region is indicated on the right.

In total, 2876 men (65.4%) underwent observation, 792 (18.0%) received active treatment, and in 728 cases (16.6%), the management strategy was unclear (Fig. 1). Among those who received active treatment, 562 (71.0%) underwent radical prostatectomy, 164 (20.7%) received radiotherapy, and 66 (8.3%) were treated with brachytherapy or focal therapy. Within the group with unknown management, 510 patients (70.1%) had therapies for bladder outlet obstruction and 218 (29.9%) were registered as undergoing “other treatments” or had unspecified treatments.

In univariable logistic regression analyses, the ORs for receiving an observational strategy were 1.08 (95% CI 0.89–1.30) for the middle SEP and 1.24 (95% CI 1.02–1.50) for the high SEP group, compared with the patients in the low SEP group (Supplementary Table 2).

**Fig. 2:**
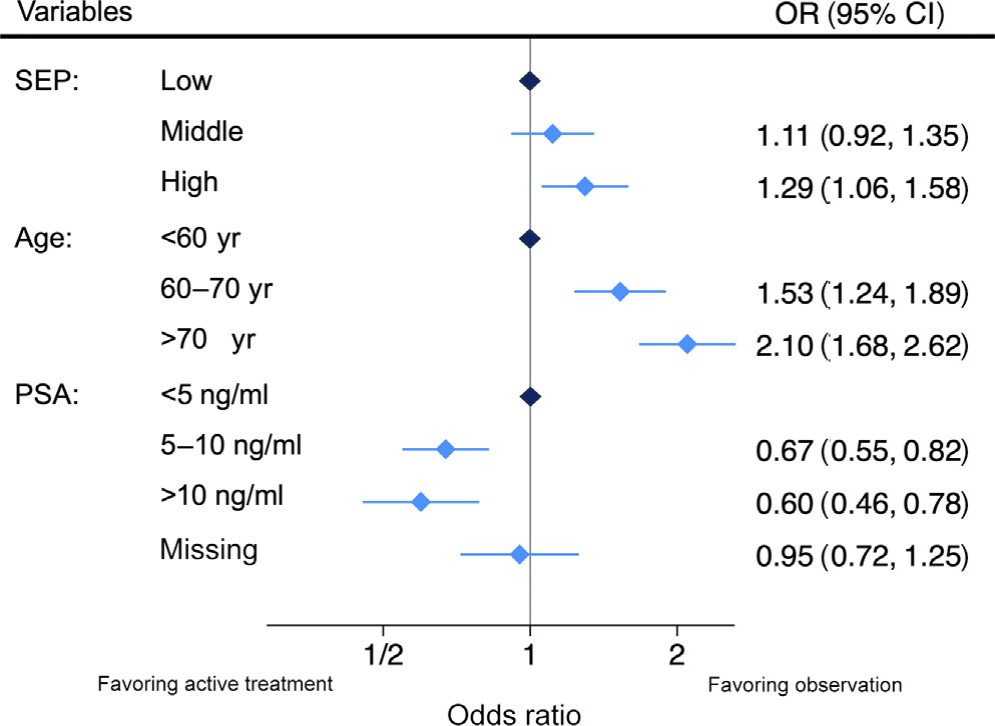
Results from the multivariable logistic regression analysis for the association between SEP and observational treatment versus active treatment. The model was adjusted for age group, PSA category, and region. CI = confidence interval; OR = odds ratio; PSA = prostate-specific antigen; SEP = socioeconomic position.

**Fig. 3:**
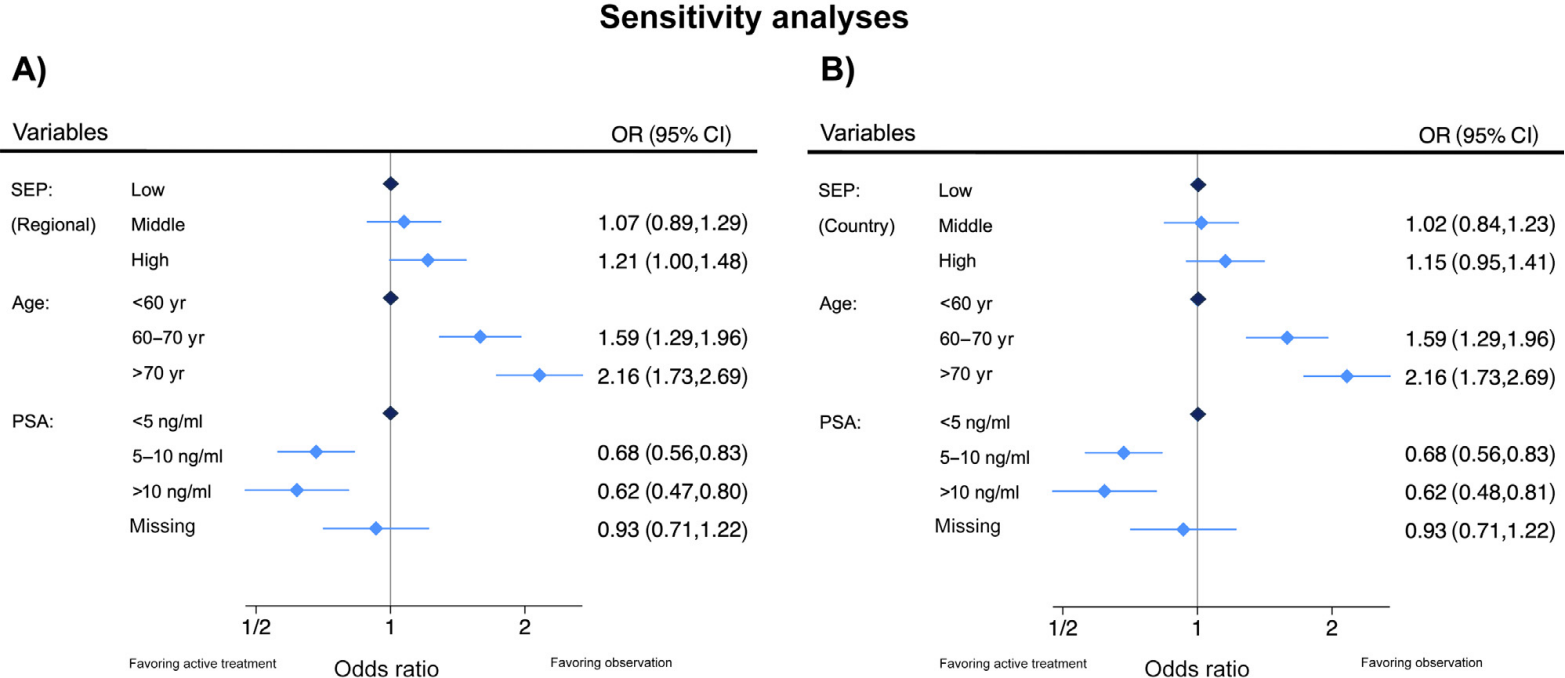
Sensitivity analysis using adapted multivariable regression models without the correction for regions. (A) The model analyzed the association of regional SEP with observational management, adjusting for age group and PSA category. (B) The model analyzed the association of SEP tertiles across the whole country with observational management, adjusting for age group and PSA category. CI = confidence interval; OR = odds ratio; PSA = prostate-specific antigen; SEP = socioeconomic position.

After adjustment in multivariable logistic regression analyses, the ORs for undergoing observation were 1.11 (95% CI: 0.92–1.35) for the middle SEP group and 1.29 (95% CI: 1.06–1.58) for the high SEP group, compared with the low SEP group. Age was also significantly associated with observational management. Compared with men younger than 60 yr, those aged 60–70 yr had an OR of 1.53 (95% CI: 1.24–1.89), and those older than 70 yr had an OR of 2.10 (95% CI: 1.68–2.62). Meanwhile, higher PSA levels were associated with reduced odds of observational management. Men with PSA levels of 5–10 ng/ml had an OR of 0.67 (95% CI: 0.55–0.82), and those with PSA >10 ng/ml had an OR of 0.60 (95% CI: 0.46–0.78), compared with those with PSA <5 ng/ml (Fig. 2 and Supplementary Table 3). In our analysis, adjusting for region had a small influence on the association between SEP and observational management, whereas age category and PSA had a negligible effect. Nevertheless, exclusion of region from the multivariable regression analysis revealed qualitatively similar results to those of the main analysis (Fig. 3A). When using SEP tertiles across the entire country (Fig. 3B), we found a weaker association of SEP with observational management, with an OR of 1.02 (95% CI: 0.84–1.23) for the middle SEP group and 1.15 (95% CI: 0.95–1.41) for the high SEP group compared with the low SEP group.

**Supplementary Fig. 1:**
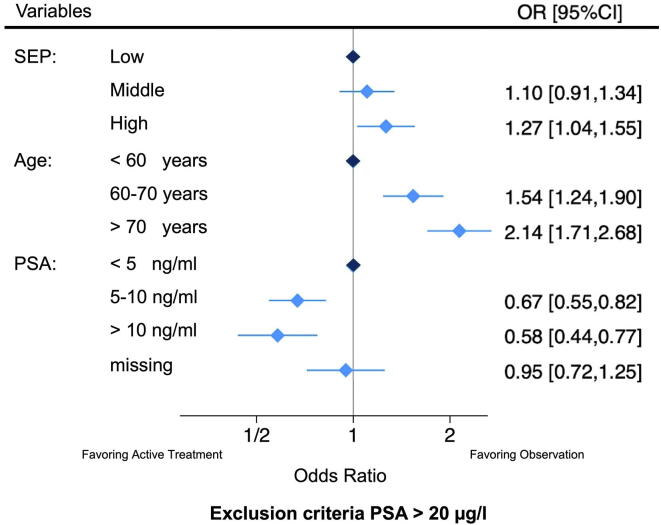
Sensitivity analysis using multivariable logistic regression for the association between SEP and observational treatment vs. active treatment, excluding patients with PSA values > 20 ng/ml. The model was adjusted for age group, PSA category, and region. Abbreviations: CI, Confidence Interval; SEP, Socioeconomic Position; PSA, Prostate-Specific Antigen; OR, Odds Ratio.

**Supplementary Fig. 2:**
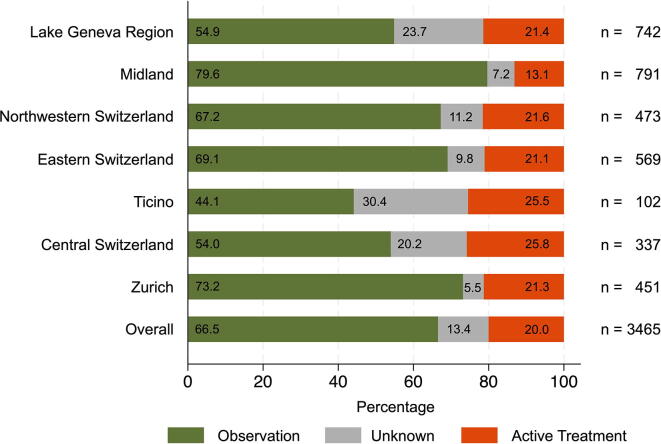
Sensitivity analysis of the distribution of chosen treatment among men with Gleason Score 6 prostate cancer across Swiss regions, excluding patients >75 years. The bars represent the proportion of patients managed with observation (green), with unknown (grey), or active treatment (red). The number of patients per region is indicated on the right.

**Supplementary Fig. 3:**
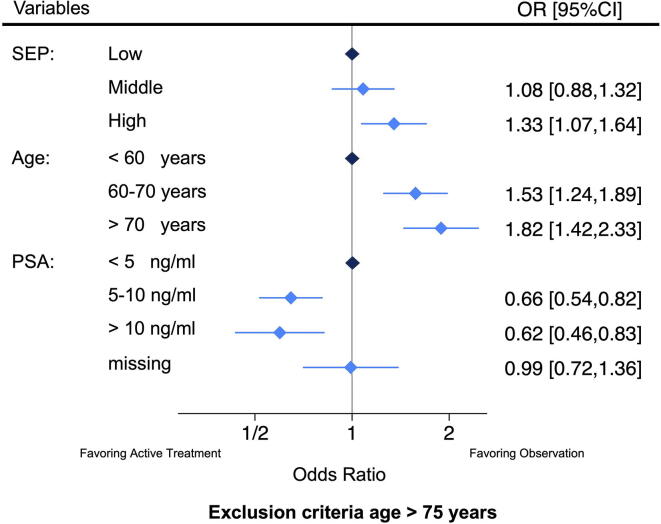
Sensitivity analysis using multivariable logistic regression for the association between SEP and observational treatment vs. active treatment, excluding patients >75 years. The model was adjusted for age group, PSA category, and region. Abbreviations: CI, Confidence Interval; SEP, Socioeconomic Position; PSA, Prostate-Specific Antigen; OR, Odds Ratio.

**Supplementary Fig. 4:**
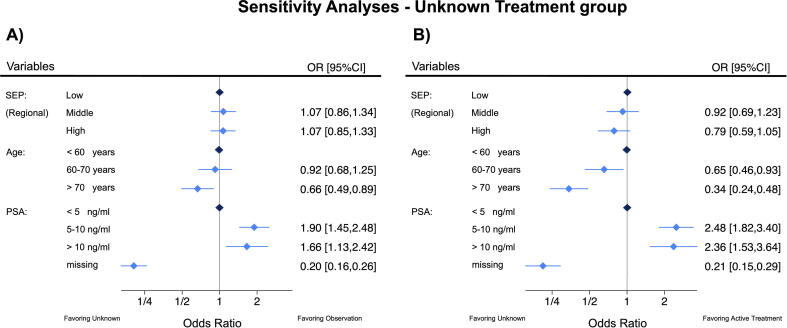
Sensitivity analyses using multivariable logistic regression for the association between SEP and unknown management (A) compared with observational management and (B) compared with active treatment. The models were adjusted for age group, PSA category, and region. Higher SEP terciles were negatively associated with unknown treatment compared with observational management, and positively associated with unknown treatment compared with active treatment. This means that the unknown treatment group had a SEP distribution lying somewhere between the active treatment group and the observational management group and is likely composed of patients belonging to either group. Patients in the unknown treatment group were older than those in the observational and active treatment groups. The associations of PSA categories are difficult to interpret, as over half of the PSA values were missing in the unknown treatment group. Abbreviations: CI, Confidence Interval; SEP, Socioeconomic Position; PSA, Prostate-Specific Antigen; OR, Odds Ratio.

In further sensitivity analyses excluding an additional 57 patients with PSA >20 ng/ml, we found a minimal impact on the overall results (Supplementary Fig. 1). Exclusion of 931 patients older than 75 yr led to a slight decrease in missing data within the cohort, accompanied by a minimal increase in relative active treatment and decrease in observation (Supplementary Fig. 2 and 3). The results of the models evaluating the association of SEP, age, and PSA with unknown treatment, compared with observational management and active treatment are reported in Supplementary Fig. 4.

## 4 Discussion

In 2020 and 2021, most men with localized low-risk PCa in Switzerland were managed with observation. These results represent a notable increase in conservative management, compared with previously available data [11]. Despite this encouraging trend, our analysis indicates that at least 18% of patients still underwent active treatment, primarily radical prostatectomy, even though observation would have been a safe primary management option with the potential to reduce treatment-associated morbidity [3]. Notably, active treatment was significantly associated with lower SEP, younger age, and higher PSA levels.

AS has been recommended as the primary management option for patients with low-risk PCa in the European Association of Urology guidelines since 2009 [15]. It has been shown to successfully delay or completely avoid local curative treatments, leading to a reduction in overtreatment as well as the risk of morbidity associated with treatment [16]. Despite this long-standing recommendation, variations in the use of AS in different settings have been observed [7], [17]. In registry-based analyses of US patients, it has been shown that a patient's higher SEP increased the likelihood of undergoing AS [18], [19]. Conversely, a recent meta-analysis combining data from studies in Australia, Canada, Sweden, and the USA found only an association between low education and nonactive treatment [20]. In contrast, our analysis showed that higher SEP within a region was significantly associated with observational treatment. Australia, Canada, and Sweden have tax-based, centralized health care systems, potentially resulting in more standardized patient pathways [21], [22], [23], [24]. Meanwhile, the US and Swiss health care systems are insurance based, with mandatory insurance in Switzerland, resulting in stronger market effects that enable patients to choose their treating physician and treatment strategy [25], [26]. More centralized health care systems tend to have a higher proportion of observational treatment [6], [8], [27], [28].

SEP could ultimately influence the chosen therapy in either direction. On the one hand, higher SEP probably improves the health literacy of individual patients, thereby preventing overtreatment due to the presence of well-informed individuals within this peer group. However, on the other hand, higher SEP could also increase the risk of overtreatment due to the financial incentives of the health care system [29]. However, specific AS incentives did not seem to lead to stronger uptake [30]. Similarly, our results do not support the notion that financial factors are a primary driver of AS adoption, as the Swiss health care system predominantly operates on a fee-for-service model, in which privately insured patients generate higher revenue through interventional procedures.

Various measures to improve the uptake of AS have been discussed. Educating both physicians and patients seems to be the key for understanding the safety and benefit of AS [31]*.* Moreover, revision of terminology, such as reclassification of Gleason 6 PCa to a label without the term “cancer,” may alleviate fear and reduce overtreatment [1], [2], [32]. Implementation of system-level strategies, including multidisciplinary case reviews and ongoing audit processes, may support improved adherence to guidelines and ensure a more standardized approach to care [33].

Our sensitivity analyses showed that the influence of SEP was somewhat smaller when the SEP terciles of the whole country were used instead of regionally adapted terciles. SEP varies substantially between different regions. As a result, a municipality in the high SEP group of a low SEP region would be in the low SEP group of another region. This means that the relative SEP of a patient within a region is a stronger predictor than the SEP at the national level.

In Switzerland, treatment coding is done regionally. In our analysis, some regions had more than a quarter of PCa cases with no recorded cancer-related treatment code, which was often after the incidental finding of PCa following bladder outlet obstruction therapy. Owing to the unclear management strategy, these cases were not classified into the active treatment or observation groups. Consequently, we deliberately chose not to perform a comparison of regions, given the substantial risk of misinterpretation.

In our analysis, a higher baseline PSA value was associated with increased odds of undergoing active treatment. Klotz et al. [16] described a higher baseline PSA as a risk factor for later upstaging during follow-up of patients under AS. Additionally, the PRIAS study found that higher baseline PSA levels were predictors of disease progression, with PSA density and number of positive baseline biopsy cores being the strongest predictors [34]. Importantly, baseline biopsies in both studies were performed before the multiparametric magnetic resonance imaging (mpMRI) era. Thus, undersampling and undergrading in the baseline biopsy was possible. Therefore, it remains unclear whether a higher PSA level continues to be a risk factor for upgrading in the era of mpMRI-guided biopsy. Most patients in our analysis underwent mpMRI-guided biopsies, as this was the standard of care in Switzerland in 2020 [35].

Multiple cohort studies demonstrated that age was an independent risk factor for upstaging in follow-up biopsies [36], [37]. Conversely, our analysis indicates that younger men had increased odds of undergoing active treatment, although AS is a safe option in young men. This seems especially relevant, since younger men have higher baseline sexual and urinary function scores and therefore have a greater potential to lose the most by immediate active treatment [38].

Our study has some limitations. No single treatment code exists in Switzerland that differentiates reliably between AS and WW. Therefore, we combined both management strategies as an observational management strategy. Swiss cantonal cancer registries document a composite Gleason score, where the Gleason score of the prostatectomy specimen replaces the Gleason score of the biopsy if a prostatectomy is performed. Hence, up- or downstaging after prostatectomy modifies the Gleason score in the NACR dataset. In Switzerland, patients from a particular region are not necessarily required to be treated at hospitals within the same area, but they are free to seek treatment in another region of Switzerland. Unfortunately, the NACR does not receive information on the region of the treatment, but only on the patient’s municipality of residence. Additionally, there were significant missing data regarding treatment decisions in certain regions, which could have influenced the results of the logistic regression. As we do not assume that the data are missing at random, we intentionally refrained from performing statistical comparisons across regions. Moreover, reliance on the Swiss-SEP precluded the analysis of the SEP individual components.

## 5 Conclusions

Most men diagnosed with localized PCa with a Gleason score of 6 in Switzerland between 2020 and 2021 underwent observational management as the primary treatment strategy. However, ∼20% of men still received active treatment for low-risk PCa. Lower SEP, younger age, and higher PSA values were identified as risk factors for active treatment in low-risk PCa setting.

  ***Author contributions*:** Thomas Paul Scherer had full access to all the data in the study and takes responsibility for the integrity of the data and the accuracy of the data analysis.

  *Study concept and design*: Scherer, Poyet.

*Acquisition of data*: Scherer, Menges, Wildisen.

*Analysis and interpretation of data*: Scherer, Menges.

*Drafting of the manuscript*: Scherer, Menges, Bieri, Kaufmann, Schmid.

*Critical revision of the manuscript for important intellectual content*: Wildisen, Staehelin.

*Statistical analysis*: Scherer, Menges.

*Obtaining funding*: None.

*Administrative, technical, or material support*: Eberli.

*Supervision*: Eberli, Rohrmann, Poyet.

*Other*: None.

  ***Financial disclosures:*** Thomas Paul Scherer certifies that all conflicts of interest, including specific financial interests and relationships and affiliations relevant to the subject matter or materials discussed in the manuscript (eg, employment/affiliation, grants or funding, consultancies, honoraria, stock ownership or options, expert testimony, royalties, or patents filed, received, or pending), are the following: None.

  ***Funding/Support and role of the sponsor*:** No funding was obtained for this study. Dominik Menges received project and salary funding for an unrelated project by the Swiss Cancer Foundation (SCF), the Dr. Arnold U. und Susanne Huggenberger-Bischoff Stiftung, and one private, not-for-profit research foundation from Switzerland requesting anonymity via the University of Zurich.

  ***Acknowledgments*:** We would like to thank the Cantonal Cancer Registries for the registration of the data used in this study, namely: Y. Bergeron (CR-FR), A. Bordoni (CR-TI), I. Curjuric and M. Adam (CR-AG), G. Defossez (CR-VD), J. Diebold (CR-LU/UR/OW/NW), S. Erny (CR-BS/BL), I. Konzelmann (CR-VS), M. Maspoli and J.L. Bulliard (CR-NE/JU), M. Mousavi (CR-SG/TG/AI/AR), A. Perren (CR-BE/SO), E. Rapiti (CR-GE), S. Rohrmann (CR-ZH/ZG/SH/SZ), and R. von Moos (CR-GR/GL). Furthermore, we thank R. Panczak, C. Berlin, M. Voorpostel, M. Zwahlen, and M. Egger for providing access to the Swiss-SEP data. We also acknowledge the National Agency for Cancer Registration (NACR) for merging the cantonal data, providing the national data, and conducting data linkage with the Swiss-SEP, which enabled the national analysis.

  ***Data sharing statement*:** Only registry data were used in this project, which is available upon reasonable request directly from the National Agency for Cancer Registration (NACR).
