# Supplementary material for: Association of Socioeconomic Position, Prostate-specific Antigen, and Age with Observation in Low-risk Prostate Cancer Patients in Switzerland
Source: Eur Urol Open Sci. 2025 Dec 30;83:198–204. doi: 10.1016/j.euros.2025.12.009 (PMC12803993; doi:10.1016/j.euros.2025.12.009)
Supplement: Supplementary Tables 1-3 [file mmc1.docx]

**Supplementary material**

**Supplementary Table 1:**

| Observation | "89.0A.09", "998“, "999" |
| --- | --- |
| Radiotherapy | "92.2“, "92.22.00", “92.24", "92.24.00", "92.24.09", “92.25.00", "92.26", "92.26.00", "92.31.10" |
| Brachytherapy | "92.27", "92.27.00", "92.29.00", "92.29.09", "92.29.3", "92.29.40", |
| Focal Therapy | “00.RA”, "60.99.11", "60.62" |
| Radical Prostatectomy  Lymphadenectomy | "60.5X.00", "60.5X.10", "60.5X.30", "60.5X.99", "60.73"  "40.3X.00", "40.3X.20", "40.3X.23", "40.3X.24", "40.3X.29", "40.53.11" |
| Bladder outlet obstruction therapies | "00.RB", "00.RK", "60.20", "60.21.12", "60.22", "60.29", "60.4", "60.61.00", "60.61.10", "60.61.11", "60.61.99", "60.69", "60.96", |
| Other Therapy | "99.BC", "00.R9", "00.9", "60.99.00", "60.99.09", "99.99" |
| Cystectomy | "57.71", "57.71.00", "57.71.11", "57.71.20", "57.49.00", "57.71.50", "57.71.51", "57.79.00" |
| Systemic treatment | “99.2R.0”, "99.2R.01", "99.2R.05", "99.2R.06", "99.2R.07" |

**Supplementary Table 1:** The registered CHOP Codes were categorized into the following treatment categories. Abbreviation: CHOP-Codes: Swiss Classification of Surgical Interventions

**Supplementary Table 2**

| **Observation** | | **OR** | **P-Value** | **[95%conf.** | **interval]** |
| --- | --- | --- | --- | --- | --- |
|  | |  |  |  |  |
| **SEP** |  |  |  |  |  |
| low |  |  |  |  |  |
| middle |  | 1.08 | 0.4 | 0.89 | 1.30 |
| high |  | 1.24 | 0.033 | 1.02 | 1.50 |
|  |  |  |  |  |  |
| **Age Category** (years) | |  |  |  |  |
| <60 |  |  |  |  |  |
| 60-70 |  | 1.53 | <0.001 | 1.25 | 1.88 |
| ≥70 |  | 2.08 | <0.001 | 1.67 | 2.57 |
|  |  |  |  |  |  |
| **PSA Category (ng/ml)** | |  |  |  |  |
| <5 |  |  |  |  |  |
| 5-10 |  | 0.72 | <0.001 | 0.60 | 0.88 |
| >10 |  | 0.70 | 0.008 | 0.54 | 0.91 |
| Missing |  | 1.09 | 0.5 | 0.83 | 1.42 |
|  |  |  |  |  |  |
| **Region** |  |  |  |  |  |
| Eastern Switzerland | |  |  |  |  |
| Lake Geneva Region | | 0.71 | 0.01 | 0.55 | 0.92 |
| Midland |  | 1.55 | 0.001 | 1.19 | 2.04 |
| Northwestern Switzerland | | 0.87 | 0.3 | 0.66 | 1.16 |
| Ticino |  | 0.46 | 0.001 | 0.29 | 0.74 |
| Central Switzerland | | 0.66 | 0.01 | 0.49 | 0.91 |
| Zurich |  | 1.02 | 0.9 | 0.76 | 1.35 |
|  |  |  |  |  |  |

**Supplementary Table 2:** Odds ratios from the univariable logistic regression analyses for observational management.

**Supplementary Table 3**

| **Observation** | | **OR** | **P-Value** | **[95%conf.** | **interval]** |
| --- | --- | --- | --- | --- | --- |
|  | |  |  |  |  |
| **SEP** |  |  |  |  |  |
| low |  |  |  |  |  |
| middle |  | 1.11 | 0.2 | 0.92 | 1.35 |
| high |  | 1.29 | 0.01 | 1.06 | 1.58 |
|  |  |  |  |  |  |
| **Age Category** (years) | |  |  |  |  |
| <60 |  |  |  |  |  |
| 60-70 |  | 1.53 | <0.001 | 1.24 | 1.89 |
| ≥70 |  | 2.10 | <0.001 | 1.68 | 2.62 |
|  |  |  |  |  |  |
| **PSA Category** (ng/ml) | |  |  |  |  |
| <5 |  |  |  |  |  |
| 5-10 |  | 0.67 | <0.001 | 0.55 | 0.82 |
| >10 |  | 0.60 | <0.001 | 0.46 | 0.78 |
| Missing |  | 0.95 | 0.7 | 0.72 | 1.25 |
|  |  |  |  |  |  |
| **Region** |  |  |  |  |  |
| Eastern Switzerland | |  |  |  |  |
| Lake Geneva Region | | 0.69 | 0.005 | 0.53 | 0.90 |
| Midland |  | 1.59 | 0.001 | 1.21 | 2.08 |
| Northwestern Switzerland | | 0.85 | 0.2 | 0.63 | 1.13 |
| Ticino |  | 0.47 | 0.002 | 0.29 | 0.76 |
| Central Switzerland | | 0.68 | 0.02 | 0.50 | 0.93 |
| Zurich |  | 0.99 | 0.9 | 0.74 | 1.32 |
|  |  |  |  |  |  |
| Constant |  | 2.84 | 0 | 2.11 | 3.80 |

**Supplementary Table 3:** Odds ratios from the multivariable logistic regression analysis for observational treatment (displayed in Figure 2).
